# Cell-cell interactions and fluctuations in the direction of motility promote directed migration of osteoblasts in direct current electrotaxis

**DOI:** 10.3389/fbioe.2022.995326

**Published:** 2022-10-06

**Authors:** Jonathan Edward Dawson, Tina Sellmann, Katrin Porath, Rainer Bader, Ursula van Rienen, Revathi Appali, Rüdiger Köhling

**Affiliations:** ^1^ Institute of General Electrical Engineering, University of Rostock, Rostock, Germany; ^2^ Department of Chemistry and Physics, Augusta University, Augusta, GA, United States; ^3^ Oscar-Langendorff-Institute of Physiology, Rostock University Medical Center, Rostock, Germany; ^4^ Department of Life, Light and Matter, Interdisciplinary Faculty, University of Rostock, Rostock, Germany; ^5^ Biomechanics and Implant Research Lab, Department of Orthopedics, Rostock University Medical Center, Rostock, Germany; ^6^ Department of Ageing of Individuals and Society, Interdisciplinary Faculty, University of Rostock, Rostock, Germany; ^7^ Center for Translational Neuroscience Research, Rostock University Medical Center, Rostock, Germany

**Keywords:** cell migration, electrotaxis, osteoblasts, computational modeling, collective migration, particle based approach

## Abstract

Under both physiological (development, regeneration) and pathological conditions (cancer metastasis), cells migrate while sensing environmental cues in the form of mechanical, chemical or electrical stimuli. In the case of bone tissue, osteoblast migration is essential in bone regeneration. Although it is known that osteoblasts respond to exogenous electric fields, the underlying mechanism of electrotactic collective movement of human osteoblasts is unclear. Here, we present a computational model that describes the osteoblast cell migration in a direct current electric field as the motion of a collection of active self-propelled particles and takes into account fluctuations in the direction of single-cell migration, finite-range cell-cell interactions, and the interaction of a cell with the external electric field. By comparing this model with *in vitro* experiments in which human primary osteoblasts are exposed to a direct current electric field of different field strengths, we show that cell-cell interactions and fluctuations in the migration direction promote anode-directed collective migration of osteoblasts.

## 1 Introduction

The response of the cell to its sensory inputs plays a crucial role in many biological processes such as embryonic development, tissue formation/regeneration, and wound healing. One of the common but crucial reactions of cells is their directed motility, where cells alter their motion in response to external stimuli. Generally, such stimuli are considered to consist of chemical (chemotaxis) or mechanical (adhesion and substrate contact; haptotaxis) mechanisms, as well as of temperature gradients (thermotaxis) or electric fields (electrotaxis) (Simpson et al., 2017), (Lara Rodriguez and Schneider, 2013), (Wu and Lin, 2011), (Zajdel et al., 2020). The latter, electrotaxis, also termed galvanotaxis, is being increasingly studied, in particular in keratinocytes and fibroblasts, since it may provide a promising strategy to foster skin wound healing (Liang et al., 2020), (Cho et al., 2018), (Lin et al., 2017), (Saltukoglu et al., 2015). Electric fields are an attractive approach for manipulating cell behavior because of their broad application across multiple cells and tissue types (Dawson et al., 2020; Gruening et al., 2021). In most cells, the direction of migration seems to be cathodal (e.g., in fibroblasts and mesenchymal stem and corneal epithelial cells (Lin et al., 2017) (Zhao et al., 2002). However, other cell types, such as adenocarcinoma cells, and bone marrow mesenchymal stem cells, show the opposite direction, i.e., anodal (Zhao et al., 2011; Lin et al., 2017). Interestingly, a reversal of directionality was reported for keratinocytes when inhibiting P2Y, a class of extracellular signal sensing surface receptors (Saltukoglu et al., 2015). We recently reported that store-operated calcium channels are pivotal for electrotaxis in human osteoblasts (Rohde et al., 2019), which interestingly migrate to the anode. Thus electrotaxis seems to depend on a variety of factors, such as cell type, environment, possibly age, and ontogenetic stage, all of which should influence cellular signaling pathway equipment.

One of the factors that has not found much consideration so far is that *in vivo*, electrotactic cell migration involves not only singular but many cells, for example, in a tissue, which collectively responds to either endogenous or exogenous electric fields. Such an electric field-dependent collective cell migration raises the question of how cell interactions, with the electric field on the one hand and the neighbouring cells on the other, shape the final migration vector. In previous modeling studies mainly reaction-diffusion based models were used (Gruler and Nuccitelli, 2000), (Schienbein and Gruler, 1993), (Camley and Rappel, 2017), in some cases cell interaction with the electrical field and chemoattractants were included (Vanegas-Acosta et al., 2012), (Wu and Lin, 2011). These approaches focused on cell migration mainly at the mean-field level and did not resolve the processes at the level of a single cell. Recently, the impact of external electrical field on single-cell motility was quantified using a Bayesian inference approach (Prescott et al., 2021). Cell-cell communication establishes a network that gives rise to many interesting behaviors, such as non-linear collective response, as observed in quorum sensing, a type of bacterial cell-cell communication (Waters and Bassler, 2005; Thurley et al., 2018). Quantitative studies have shown that collective cell migration in epithelial structures is an emergent phenomenon that cannot be explained without considering cell-cell interactions (Barton et al., 2017; Henkes et al., 2020). Self-propelled particle-based models have been widely used to study collective behavior in cell migration in tissues (Szabó et al., 2006; Trepat et al., 2009). While the inclusion of cell-cell interactions in models seems to be natural in the case of high-density tissue culture, where cells adhere to each other and thus exert a pulling force on the neighboring cells, for example, in epithelial wound healing (Brugués et al., 2014), (Cohen et al., 2014), (Zajdel et al., 2020), the rules governing such an interaction in a system of isolated cells, such as *in vitro* cell culture, remains ambiguous. Three-dimensional multi-cell models based on the finite element method computationally studied the influence of multiple signals, such as the substrate stiffness, chemical gradient, and the electric field on the migratory behavior of cell collective (Mousavi et al., 2013, 2014; Mousavi and Doweidar, 2015; Urdeitx and Doweidar, 2020).

Here, we present a computational model to study the motility and collective migration of osteoblast cells stimulated by direct current (DC) electric field. We compare our model with quantitative experiments and test the hypothesis that cell-cell interactions shape the total vector by re-analyzing data on an individual cell basis from our recent study on osteoblast migration mechanisms in DC electric field (Rohde et al., 2019). Our modeling framework incorporates two-dimensional motion of single cells on a substrate, fluctuations in the direction of cell migration, and key cell interactions. We computationally explore our model to study the influence of two distinct biologically relevant model parameters on electrotactic migration of osteoblasts.

## 2 Materials and methods

In this study, data on cell migration of human osteoblasts under DC-electrical stimulation were re-analyzed using a previous set of experiments (Rohde et al., 2019). Cell cultivation and stimulation methods are detailed in this paper and given in brief below:

### 2.1 Cell culture

Human osteoblasts were isolated from femoral heads of patients (*n* = 14) undergoing a total hip replacement. Patients gave consent and the study was approved by the local ethics committee (permit A 2010–10). Osteoblasts were isolated from cancellous bone as previously described (Lochner et al., 2011). Isolated cells were cultured in Dulbecco’s Modified Eagle Medium (Pan Biotech, Aidenbach, Germany) supplemented with 10% fetal calf serum, 1% amphotericin B, 1% penicillin-strepto-mycin and 1% hepes- buffer under standard cell culture conditions (5% CO_2_ and 37°C). Ascorbic acid (50 *μ*g/ml), *β*-glycerophosphate (10 mM), and dexamethasone (100 nM) (Sigma Aldrich, St. Louis, MO, United States) were added to cell culture medium to maintain osteoblast phenotype. For cell migration experiments cells in passage three were used.

### 2.2 Direct current electrical stimulation chamber and experimental procedure

To study migration of osteoblasts in electric fields, we used a two-part stimulation chamber described in (Rohde et al., 2019). Before each use, both chamber parts were cleaned with 70% ethanol, washed with a mild detergent and rinsed extensively with distilled water before steam sterilization. Coverslips (24 × 50 mm) for seeding osteoblast cultures were coated with rat tail collagen (Advanced Biomatrix, San Diego, CA, United States) by incubation of 50 *μ*m/ml rat tail collagen diluted in sterile 0.1% acetic acid for 1 h. Coverslips were positioned in a groove in the upper chamber part and edges sealed with silicon paste (Korasilone, Obermeier GmbH, Bad Berleburg, Germany). Upper and lower chamber parts were bolted by 12 screws to ensure tight contact and prevent leakage and chambers were exposed to UV light for sterilization. After this sterilisation treatment, remaining solution was aspired and coverslips were washed twice with phosphate buffered saline (Biochrom, Berlin, Germany) before cell seeding. A total of 2 × 10^3^ osteoblasts were seeded per chamber and cells were allowed to adhere for 30 min. Afterwards, coverslips were washed twice with medium to remove non-adherent cells. Chambers were then sealed with a top coverglass, and silicon paste and cells accommodated to chamber overnight. For DC-stimulation, silver/silver chloride electrodes were placed into outer reservoirs separated from cell area to avoid electrochemical reactions within the tissue chamber. Current was conducted to the cell chamber using agar bridges (silicon tubes, length 120 mm, inner diameter 5 mm) consisting of 2% agarose (TopVision agarose, ThermoScientific, Waltham, MA, United States) in Ringer’s solution (Braun, Melsungen, Germany). Current was applied to the electrodes for 7 h *via* crocodile clamps using a DC power supply (Standard Power Pack P25, Biometra, Göttingen, Germany). To maintain constant stimulation, voltage was measured directly at the borders of the cell area (electrode distance 24 mm) using a multimeter (Voltcraft VC220, Conrad Electronic, Wollerau, Switzerland) and adjusted during the experiments. Each of the experiments was conducted with one cell culture being divided to obtain a sham stimulation group as control, and a DC-stimulation group for the respective field strength used. Electric field strengths were 160, 300, 360, 426 and 436 V/m.

### 2.3 Migration analysis

**FIGURE 1:**
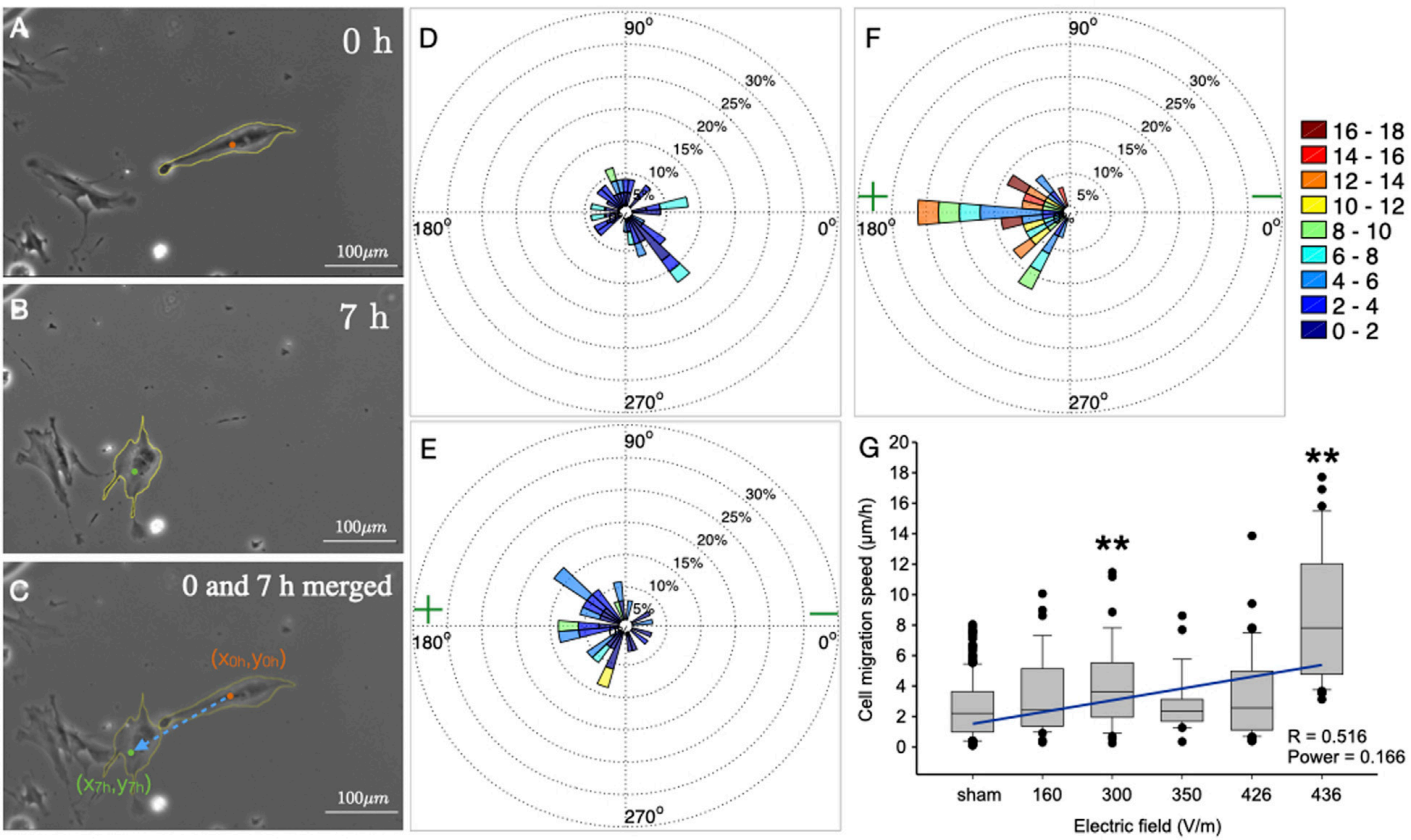
Single-cell analysis of migration of osteoblasts in a DC electric field. **(A–C)** Photomicrographs of osteoblasts in stimulating chamber. Cell boundaries were manually outlined as depicted (yellow coastline). **(A)** Position of the cells before DC-stimulation. **(B)** Position of the same cells after 7 h DC-stimulation (436 V/m). **(C)** Overlay of **(A,B)**. The dashed blue arrow shows the displacement of the centroid of the same cell before (orange dot) and after (green dot) 7 h DC-stimulation. **(D–F)** Polar plots showing the velocity of cell migration in the cases of sham **(D)**, and DC stimulation of electric field strength 160 V/m, **(E)**, and 436 V **(F)**. Each polar plot has been divided into 36 sectors, and data of cells migrating within each 10° sector are cumulated. The speed range is colour coded (in *μ*m/h) as shown in the colourbar. The relative sector lengths denote the percentage of cells migrating at a certain speed range. **(G)** Box and whisker plot of medians (horizontal lines) of cell migration speed vs. electric field strength. Whiskers denote 25–75 percentiles of data distribution. Dots show data lying outside these percentiles. Numbers of cells for each experiment are: 177, 34, 35, 26, 43, 33 for sham, and 160, 300, 350, 426 and 436 V/m, respectively. Both at 300 V/m, and at the maximum strength of 436 V/m, the speed is significantly higher than under all other conditions (Asterisks denote *p* < 0.001; ANOVA on ranks, all-pairwise comparisons using Dunn’s test).

For the analysis, all cells from the sham groups were pooled as one control. Thus, a total of *n* = 177 (sham), 34 (160 V/m), 35 (300 V/m), 26 (360 V/m), 43 (426 V/m) and 33 (436 V/m) cells were analysed. For this, photographs were taken at eight fields of view evenly distributed over the cell area at beginning (Figure 1A) and end time points (Figure 1B) with a Leica DMI 6000 and LAS X software during the 7-hour (h) stimulation, or sham stimulation, procedure. The pairs of photographs were then aligned manually, and merged, taking external markers as reference points (Figure 1C). To quantify migration within the electric field, segmentation of the cell shape, including cell extensions, was performed manually using Image J software (NIH) for each cell that could be identified in both the time points (see yellow coastlines in Figure 1C), i.e., 0 h and 7 h after DC stimulation. The segmentation procedure yielded, among other features, the coordinates of the cell centroid. Using the coordinates of the cell centroid at these two time points, i.e., 0 h and 7 h, the distance and direction of migration was calculated for each cell. The migration distance was defined as $d=\sqrt{{\left(X1-X2\right)}^{2}+{\left(Y1-Y2\right)}^{2}}$ , where *X*1, *Y*1 and *X*2, *Y*2 represent the coordinates of the cell centroid at 0 h and 7 h after DC stimulation, respectively Figure 1C. The migration angle was defined as $\tan^{-1}\left(\frac{Y2-Y1}{X2-X1}\right)$ . Using the distance and direction of cell migration we obtained a migration plot for each case of electrical stimulation, which could be depicted in a polar coordinate system, as shown in. The anode in the polar plots of DC stimulated experiments is located at 180° angle. For better comparison of all experiments, we binned the migration angles into 36 sectors of 10° each, and classified migration speeds in a scoring system. Thus, the migration angle was calculated starting from the original cell position, and angles were assigned to the 36 sectors, where sector 10–18 (90–180°) and 18–26 (180–270°) represent anode-directed migration, while sectors 1–9 and 27–36 (0–90 and 270–360°) represent cathode-directed cell migration. To construct polar plots (Figures 1D,E) illustrating both migration direction and velocity, the migration speed of single cells was colour coded from 0 to 18 *μ*m/h in nine groups of 3 *μ*m/h bins. The relative sector lengths denote the percentage of cells migrating at a certain speed range.

## 3 Results

### 3.1 *In vitro* direct current stimulation of human osteoblasts

In the experimental part of this study, we exposed human osteoblasts to DC electric fields for 7 h at different stimulation strengths and matched each of these experiments with a sham-stimulated, control group treated identically, save the DC stimulation. For the analysis of the migration behaviour, we selected adherent cells in the stimulation chambers which could be identified clearly at starting and end time points of the experiment, and did not form clusters precluding the outlining of their boundaries (Figures 1A–C). Using photographs of several fields of vision in each chamber, 1–4 cells could be traced in this way per field of vision position, totalling n = 177 cells (sham stimulation), as well as *n* = 34 cells (at 160 V/m), *n* = 35 cells (at 300 V/m), *n* = 26 (at 360 V/m), *n* = 43 (at 426 V/m) and *n* = 33 (at 436 V/m). The center position coordinates were determined for each cell tracked between the two time points, and this was used to calculate the effective magnitude and direction of the cell velocity.

As one can notice in the original photographs of one typical cell from the experiment using 436 V/m stimulation, the cells move, in this case anodally, and at the same time change their shape within the 7 h stimulation (Figures 1A–C). While we did not analyse shape changes any further in this study, we took them into consideration for determining the centroids of the cells (colored dots in Figure 1C), which we used as markers to determine the net movement (blue dashed arrow in Figure 1C).

Comparing cell migration velocities (plotted as sectors of polar plots) without stimulation (Figure 1D), to those with weak (160 V/m; Figure 1E) or strong stimulation (436 V/m; Figure 1F), one can appreciate that the directionality of migration shifts with increasing field strength from random, covering all sectors of the plot (Figure 1D) to exclusively anodal, covering only the anodal sectors (Figure 1F). At the same time, also the speed of the cells appears to shift from lower speeds with a maximum of 8–10 *μ*m/h (green hues in Figure 1D corresponding to ∼2.5% of the cells) to a maximum of 16–18 *μ*m/h (red sectors in Figure 1F corresponding to ∼12% of the cells). To address the question of a possible correlation of speed and field strength, we quantified the cell migration speed of all cells in all experiments under different stimulation strengths. As shown in Figure 1G, migration speed under DC-stimulation is significantly different from sham stimulation conditions only at 300 V/m and 436 V/m (p < 0.001, ANOVA on ranks with Dunn’s all-pairwise comparisons). We further performed a linear regression on the cumulative data for all the field strengths (blue line in Figure 1G) which showed a weak correlation between field strength and migration speed, with a regression coefficient of *R* = 0.516.

### 3.2 Modeling electrotactic migration of osteoblast cells

**FIGURE 2:**
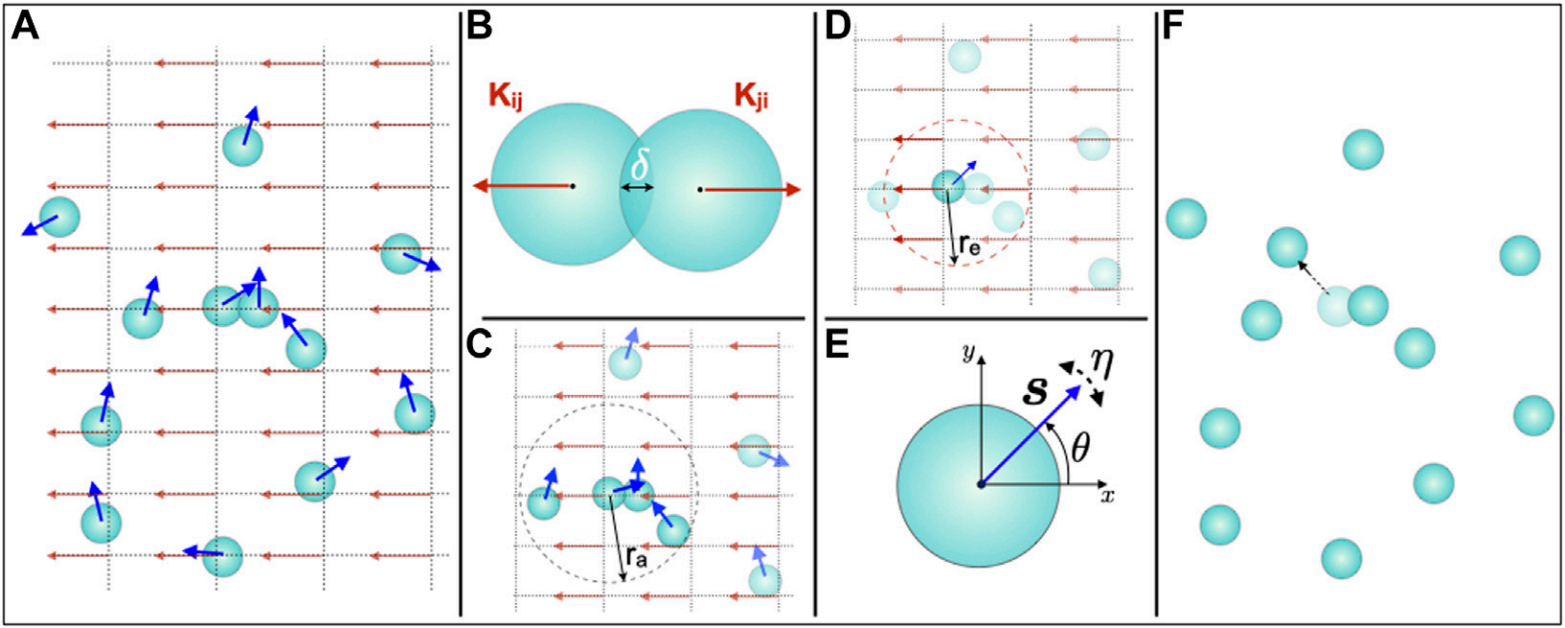
Computational model for osteoblast cell migration in an external DC electric field. **(A)** Osteoblasts in the cell culture chamber exposed to the DC electric field are modelled as active particles (light blue coloured disks) of radius *R*, and, their motion at any time *t* is described by the velocity $v_{i}^{t}$ (*i* is the index of the cell). The dark blue colored arrows laid over the circular disks are the unit vectors $s_{i}^{t}$ of the cell velocity with the magnitude *v*_0_ at time *t*. The model takes into account both cell-cell and cell-electric field interactions. These interactions can influence the cell velocity. Cell-cell interactions involve finite-volume exclusion and the alignment of migration direction. **(B)** When two cells overlap, cell *i* experiences a displacing force *K*_*ij*_ from each of its neighboring cells *j*, where *i* and *j* are the cell indices. The magnitude of such a force is proportional to the degree of overlap *δ*. **(C)** The migration direction of a cell can be influenced by its neighboring cells located within the radius *r*_*a*_, taken from the cell’s center. Such an interaction re-orients the migration direction of a cell to the average direction of migration of neighboring cells. **(D)** Each cell also experiences a force due to the electric field. The electric field is defined on a discrete two-dimensional grid underlying the space in which the cells move. The cell experiences the average force from the electric field at all the grid points that lie within the radius *r*_*e*_, taken from the cell’s center. **(E)** The limited precision in cellular sensing of directional alignment is captured by an angular white noise term whose strength is given by *η*. **(F)** The resultant direction *θ* of cell migration is the cumulative effect of the cellular interactions described in B-E. At each time step, the model calculates and updates the position of each cell (shown by a dotted arrow for the cell under consideration) for the next time step.

Our computational model simulates the *in vitro* motility behaviour of individual cells that are subject to external DC electric field, (Figure 2A). The main components of our model are (i) the ability of the cells to interact with the other cells, (Figures 2B,C), and, (ii) the ability of the cells to interact with the external electric field, (Figure 2D). Cell-cell interaction involves two types of forces: short-range repulsive forces and the alignment of the direction of motion with the cells’ local neighbors, (Figures 2B,C). Through soft-core repulsion, the force at short distances ensures that cells do not overlap. We also include in our model the influence of each cell’s local neighbors on the direction of its migration. Such cell-to-cell interactions certainly play a role in high-density tissue culture *via* cell-cell contacts. However, since mechanical or signaling cues are at least conceivable also in 2D cell cultures without direct cell contacts, we introduce this factor in the model to study the possible role of such interactions in our experiments. Finally, we also consider the interaction of cells with the applied DC electric field, (Figure 2D). We also include angular noise that describes the uncertainty that a cell experiences in directional sensing during migration, (Figure 2E).

#### 3.2.1 The cell

We describe each cell as a circular disk of radius *R* = 1 exhibiting overdamped dynamics in two dimensions (2D) with an active speed of *v*_0_. The state of each cell *i* is characterized at time *t* by its position $r_{i}^{t}$ , described through the coordinates $\left(x_{i}^{t},y_{i}^{t}\right)$ , and its migration velocity $v_{i}^{t}=v_{0}s_{i}^{t}$ , where, *v*_0_ is the cell migration speed and $s_{i}^{t}=\left(\cos\theta_{i}^{t},\sin\theta_{i}^{t}\right)$ is the unit vector representing the direction of cell migration, with *θ* being the angle that the cell makes with the horizontal axis of the laboratory frame. The direction *θ* that a cell takes at the next time step depends not only on its direction of motion in the immediately preceding time step, but also on the forces acting on the cell. The total force acting on the cell *i* results from cell-cell interactions and cell interaction with the applied DC electric field. These forces are discussed in more detail in the following sections.

#### 3.2.2 Cell-cell interactions

We consider two types of cell-cell interactions in our model, one due to finite-volume exclusion and the other resulting in the alignment of a cell’s migration direction with its neighbors. Each cell is assumed to occupy a finite area in the cell culture medium in which it is placed. In order to ensure that the cells do not overlap, we include, at each time *t*, a repulsive force ***K***_*ij*_ that is proportional to the degree of overlap between any two cells and is given by,

$$K_{ij}=k\left(2R-r_{ij}^{t}\right){\hat{r}}_{ij}^{t}$$ (1)

where, $r_{ij}^{t}$ is the euclidean distance between the center of two cells *i* and *j* at time *t* and is calculated as $\sqrt{{\left(x_{i}^{t}-x_{j}^{t}\right)}^{2}+{\left(y_{i}^{t}-y_{j}^{t}\right)}^{2}}$ . ${\hat{r}}_{ij}^{t}$ is the unit vector pointing from the center of cell *i* to the center of cell *j*. The total repulsive force acting on cell *i* at time *t*, denoted by ${F_{i}}^{t}$ is the sum of all the pairwise repulsive interactions between cell *i* and its nearest overlapping neighbors *j*,

$$F_{i}^{t}=\underset{|r_{j}^{t}-r_{i}^{t}|<2R}{\sum }K_{ij}.$$ (2)

#### 3.2.3 Cell-electric field interaction

The electric field in this model is defined on a regular square lattice underlying the domain in which the cells are migrating. Each grid point *k* whose position ***q***_*k*_ is specified by its coordinates (*m*_*k*_, *n*_*k*_). The electric field at the grid point ***q***_*k*_ located at (*m*_*k*_, *n*_*k*_) is characterized by the vector ***E***_*k*_ = *E*_0_ ***e***_*k*_, where, ***e***_*k*_ = (cos Θ_*k*_, sin Θ_*k*_) is the unit vector representing the orientation of the electric field, and, *E*_0_ is the magnitude of the electric field which corresponds to the field strength of the electrical stimulation in the experiments. Θ_*k*_ is the angle that the electric field vector at (*m*_*k*_, *n*_*k*_) makes with the horizontal *x* − axis. Due to cell spreading on the substrate, the electric field sensors on the cell membrane relocate and are distributed over a finite region in the motility domain of each individual cell. Therefore, we assume that the contribution to the total electrical force acting on a cell comes from the electric field at all the grid points within a region that the cell effectively covers due to cell spreading. We denote the extent of this region by radius *r*_*e*_ taken from the center of each cell *i*. Thus, the electric field that the cell *i* experiences is the sum total of the electric field at all the grid points that lie in the region within the radius *r*_*e*_ from the center of cell *i* and is given by,

$$D_{i}=\underset{|r_{i}^{t}-q_{k}|<r_{e}}{\sum }E_{k}.$$ (3)

Assuming that the cell motion is described by overdamped dynamics, the position of each cell *i* is updated in the next time step, after calculating the resultant displacement and angle *θ* due to all the interactions, including the directional alignment with the cells’ neighbour, by the following scheme:

$$r_{i}^{t+1}=r_{i}^{t}+\left(v_{i}^{t}+\nu F_{i}^{t}+\mu D_{i}\right),$$ (4)

where, *ν* is a friction factor that is associated with the cell-substrate interaction, *μ* is the effective electrical mobility of the cell. The time step is equal to 1.

Finally, in addition to the various forces acting on a cell, as described above, the cell in this model also undergoes directional alignment with its proximal neighbours. The directional alignment of a cell with its neighbours influences the direction of the cell’s motility and is given by,

$$\theta_{i}^{t+1}=\arg\left[\underset{|r_{i}\left(t\right)-r_{j}\left(t\right)|<r_{a}}{\sum }s_{j}^{t}\right]+\eta \xi_{i}^{t}.$$ (5)

The directional alignment in this model is only hampered by an angular white noise uniformly distributed in $\left[-\frac{\pi }{2},+\frac{\pi }{2}\right]$ with $\left\langle \xi_{i}^{t}\right\rangle =0$ and $\left\langle \xi_{i}^{t}\xi_{j}^{t^{\prime }}\right\rangle ∼\delta_{ij}\delta_{tt^{\prime }}$ and whose strength is given by *η*. arg [***c***] in the first term in Eq. 5 refers to the angle associated to the vector ***c*** if this is expressed in polar coordinates, and the sum is taken over all particles *j* within distance *r*_*a*_ of cell *i* (including *i* itself).

#### 3.2.4 Simulation details

We simulate the motility behavior of *N* = 35 cells, as in our experiments, there are approximately 30–40 cells in a single field of view. Cells are initially randomly distributed in a circular region within the spatial domain representing the stimulation chamber. Osteoblast cells are roughly 100 *μm* in diameter, considering all cells extensions, and, we use this to define the cell radius *R*, which is one length unit in our simulations. The cell radius *R* is assumed to be the basic length scale in these simulations. Time steps are separated by Δ*t* which is set to 1. The parameters of the model and their values used in these simulations are listed in Table 1. The active speed of cells is 0.1*R* per time step.

**TABLE 1:** List of all the model parameters used in simulations, their notation, description and value (dimensionless).

Parameter	Description	Value
*R*	cell radius	1
*v*_0_	active cell speed	0.1
*k*	repulsive force constant	0.3
*ν*	friction factor	0.1
*μ*	cell electrical mobility	0.04
*η*	noise strength	0.05
*r*_*a*_	distance over which directional alignment occurs	2
*r*_*e*_	distance up to which electric field is sensed by the cell	2
$\bar{D}$	strength of electric field (corresponding to 160 V/m)	0.014

The time parameters in our simulations are scaled such that the speed of the unstimulated cells corresponds to the average cell speed in the experimental sham case, which is ∼2 − 3*μ*m/h. The magnitude of the electric field *E*_0_ = 0.014 corresponds to 160 V/m in experiments and is chosen such that the cell speed, in this case, is approximately equal to the experimental value, i.e., ∼4μm/h . The magnitude of the electric field corresponding to 436 V/m in experiments is obtained by simply multiplying 0.014 by the factor 2.75, as 436/160 = 2.75. At the start of each simulation, we specify the initial positions *x*_*i*_ (*t* = 0), *y*_*i*_ (*t* = 0), initial speed *v*_*i*_ (*t* = 0) and the velocity direction *θ*_*i*_ (*t* = 0) of each cell *i*. Initially, the cells are placed at random locations within the domain. The initial velocity direction is also distributed randomly in the range [0,2*π*]. At each time step for each cell, we identify cells that are less than a distance of 2*R* apart. We developed a distance sorting algorithm to efficiently provide a list of nearest neighbors in contact with each cell for use in the simulations. From this we calculate the total repulsive force due to volume exclusion acting on each cell from all of its neighbouring cells, as given by Eqs. 1, 2. Using the same distance sorting algorithm, we also determine all the grid points of the underlying grid, on which the electric field is defined, that lie within a radius *r*_*e*_ of each cell and calculate the net electric field, denoted by ***D***_*i*_ as given by Eq. 3. This constitutes the net force due to the electric field ***D***_*i*_ acting on each cell *i*. Experiments show that the osteoblast cell migration is anode-directed. We incorporate anode-directed motility of individual cells into our model by assigning a polarity *p*, which can assume the value + 1 (cathode-directed) or − 1 (anode-directed), to the net electrical force experienced by the cell as, $D_{i}=p\sum_{|r_{i}^{t}-q_{k}|<r_{e}}E_{k}\;$ . In addition, we also determine for each cell all its neighbouring cells that are located within the radius *r*_*a*_, and calculate the net direction of velocity. The direction of velocity of each cell is then updated by its net direction, to which a weak noise *η* = 0.05 is added, as given by Eq. 5. Finally, the positions of each cell are then updated using the Eq. 4.

#### 3.2.5 Migratory behavior of osteoblasts in direct current electrical field

To study the influence of an externally applied DC electrical field on human osteoblast cell migratory behavior, we simulated *N* = 35 migrating cells with and without DC electrical stimulation for 140 time steps. We developed the computational model in (version 2021b) MATLAB (2021), wherein we described the migration dynamics of each cell by the equations of motion Eqs. 4, 5. We use periodic boundary conditions.

**FIGURE 3:**
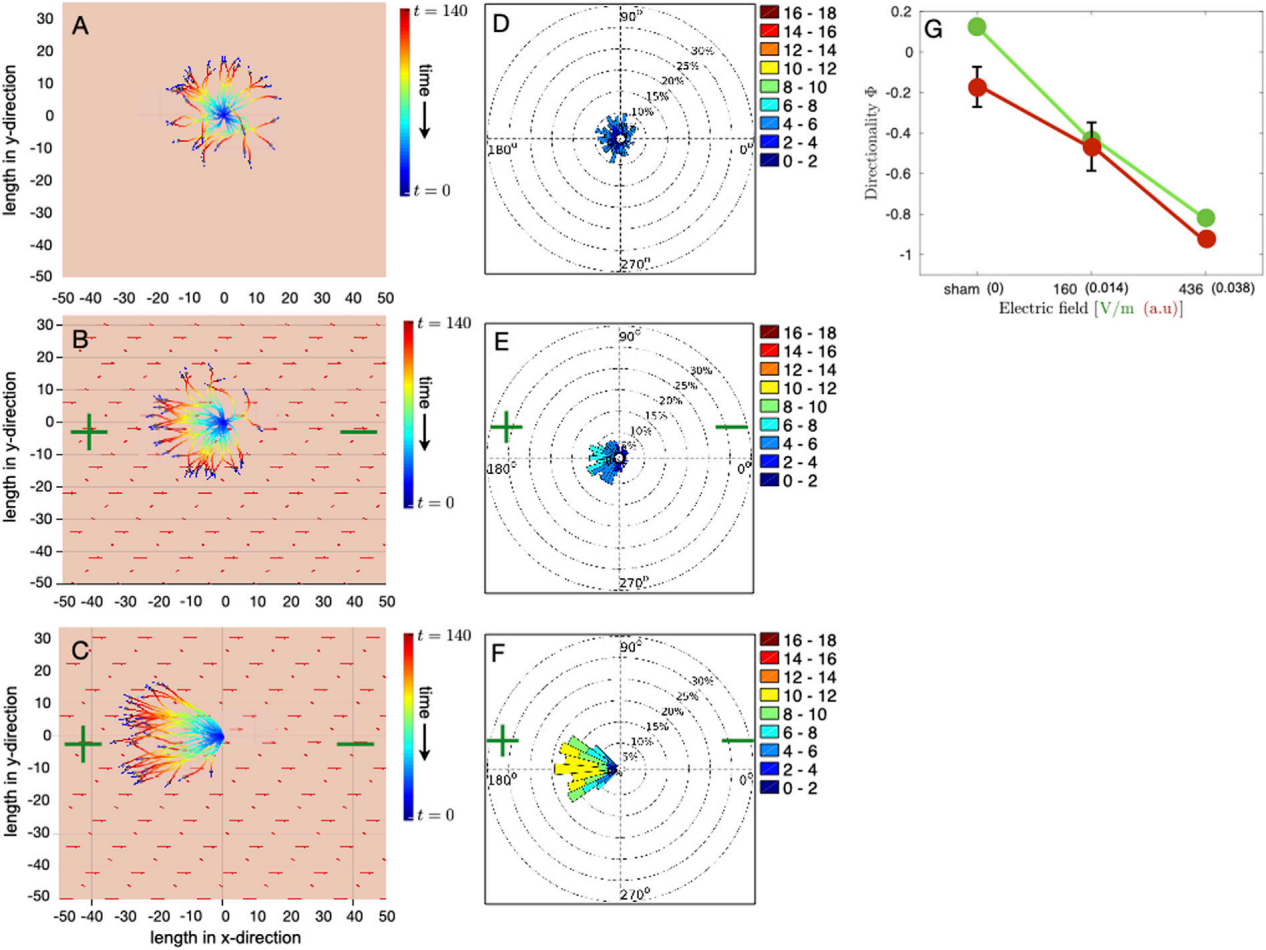
Simulation of osteoblast cell migration in DC electrical field. Each simulation consists of 35 cells, initially randomly distributed in a circular region around the center of the domain of size 50 × 50. Osteoblasts are modeled as light gray colored circular disks of radius *R* = 1 with initial migration velocities, which are randomly oriented in direction, represented by dark blue arrows. The polarity of the DC electric field is denoted by green colored plus and minus symbols, and red arrows represent the electric field vector. The model is simulated for 140 time steps. **(A–C)** Final positions and trajectories of individual cells in the case of **(A)** unstimulated cells and DC electrical stimulation of magnitude 0.014 and 0.038, shown in **(B,C)**, respectively. Cell positions are adjusted such that all the trajectories originate from *x* = 0 and *y* = 0 at *t* = 0. Color gradient of the trajectory represents time evolution, with blue color for the start of the simulation and red color for the last time point. A dimensionless value of 0.038 for the magnitude of the electric field in simulations corresponds to the maximum electric field strength of 436 V/m in experiments. **(D–F)** Polar plots show the effective velocity of cell migration, taking into account only the initial and the final time step. **(D)** shows the polar plot for the unstimulated case, whereas **(E,F)** present polar plots for 160 V/m and 436 V/m, respectively. Each polar plot is a cumulate of data from 10 separate simulation runs with random initial conditions and where each simulation consists of 35 cells. **(G)** Comparison of the directionality order parameter Φ obtained from the simulations (red color filled circles connected by red lines) with experiments (green color filled circles connected by green lines) corresponding to the three different cases, i.e., no stimulation, stimulation with field strength 160 V/m and 436 V/m, respectively. Each value of directionality obtained from simulations averages ten separate simulation runs. Error bars in simulation data show the standard deviation in Φ.

We have verified, through multiple runs of the simulations, that our results are qualitatively invariant of random initial conditions and stochastic angular fluctuations in the simulations [(see Supplementary Material)]. Figure 3 left column shows the position of all the cells at the final time step (gray circles with arrows) as well as their trajectories at each time step, in the case of no electrical stimulation, Figure 3A, and in the case of DC stimulation with the magnitude of the electric field being 0.014 (160 V/m) and 0.038 (436 V/m), Figures 3B,C, respectively. The magnitude 0.038 of the electric field in simulations corresponds to the maximum field strength of the electrical stimulation in experiments, i.e., 436 V/m, Figure 1G. The velocity of cell migration, calculated from the initial and the final time step, is shown in Figures 3D–F (right column) as polar plots for the case without electrical stimulation, Figure 3D, and with electrical stimulation of different magnitudes of electric field, i.e., 0.014 and 0.038, Figures 3E,F, respectively. Each polar plot shown in Figures 3D–F, is the cumulate of ten separate runs of the simulation. The initial velocity of each cell and the noise in the direction of cell velocity at each time step are random, and this renders robustness to the results of polar plot distributions.

In the absence of electrical stimulation, the cells move, as expected, in all directions, Figure 3A. Trajectories of individual cells show that, over time, all the cells collectively explore the space isotropically, Figures 3A,B feature that is also reflected in the polar plots, 3d. The mean cell speed in this case is ∼3μ m/h. However, when a DC electrical field of magnitude 0.014, which corresponds to 160 V/m, is applied, the cells start exhibiting a directional migration towards the anode 3b. The individual cell trajectory plot shows that although the final position of the majority of the cells is towards the anode, few cells still migrate towards the cathode, albeit at much shorter distances than the anodally migrated cells, Figure 3B. The polar plot, showing the effective cell migration velocity, clearly presents the modulation of the direction of migration by an external field, Figure 3E. Following the trajectories of individual cells also reveals that cell migration is not instantaneously switched in the direction of the anode. Cells respond to the applied electrical field by gradually changing the direction of their migration. Initially, most of the cells move randomly. However, at later times, under the influence of the electric field they gradually turn towards the anode. This delayed response in eventual anode-directed motility of cells is because the electrotactic migration speed *μ* ***D***, where *μ* is the cell mobility, is much weaker than the active cell migration speed ***v***. Cells display a highly directional motion towards the anode with increasing strength of the electric field *E*_0_, Figure 3C. Cell migration, in this case, shows a much faster re-orientation and more persistent motion towards anode 3c. Figure 3F shows that not only the direction of the motion but the effective speed of cell migration is also influenced by increasing the strength of the electric field. The maximum cell speed in this case even reaches up to 10–12 *μ*m/h, Figure 3F.

To better quantify the changes in the collective cell migratory behavior, we calculate, in both experiments and simulations, the directionality order parameter Φ, which reflects how well cell movements have aligned with the electric field and directed towards the anode and is given by,

$$\Phi =\frac{1}{N}\underset{i}{\sum }\cos\left(\theta_{i}\right)$$ (6)

where *N* is the total number of cells, and the sum is over the cosine of the migration direction of individual cells *θ*_*i*_. Since the electric field is aligned along the *x* − axis, we use the cosine component of the cell velocity to evaluate how well the cells are aligned with the external electric field. Φ can vary between 1 (perfectly aligned towards the cathode) and −1 (perfectly aligned towards the anode), and Φ ≃ 0 corresponds to both random isotropic cell movement and movement perfectly orthogonal to the direction of the applied electric field. Our results show that for the listed choice of parameters, as given in Table 1, the directionality order parameter Φ obtained from the model simulation matches very closely with the experiments (Figure 3G). Our results shown in Figure 3 reproduce the following experimental observations: (i) in the absence of electrical stimulation, which corresponds to the experimental sham case, directional migration of cells is not observed, i.e., cells do not move in a preferred direction and are not aligned with the external electric field, (ii) alignment of the direction of cell migration with the external electric field and directed towards anode depends on the strength of the applied electrical field, (iii) the effective cell velocity increases with the electric field strength.

#### 3.2.6 Influence of radius of alignment and cell number density on cell migration

**FIGURE 4:**
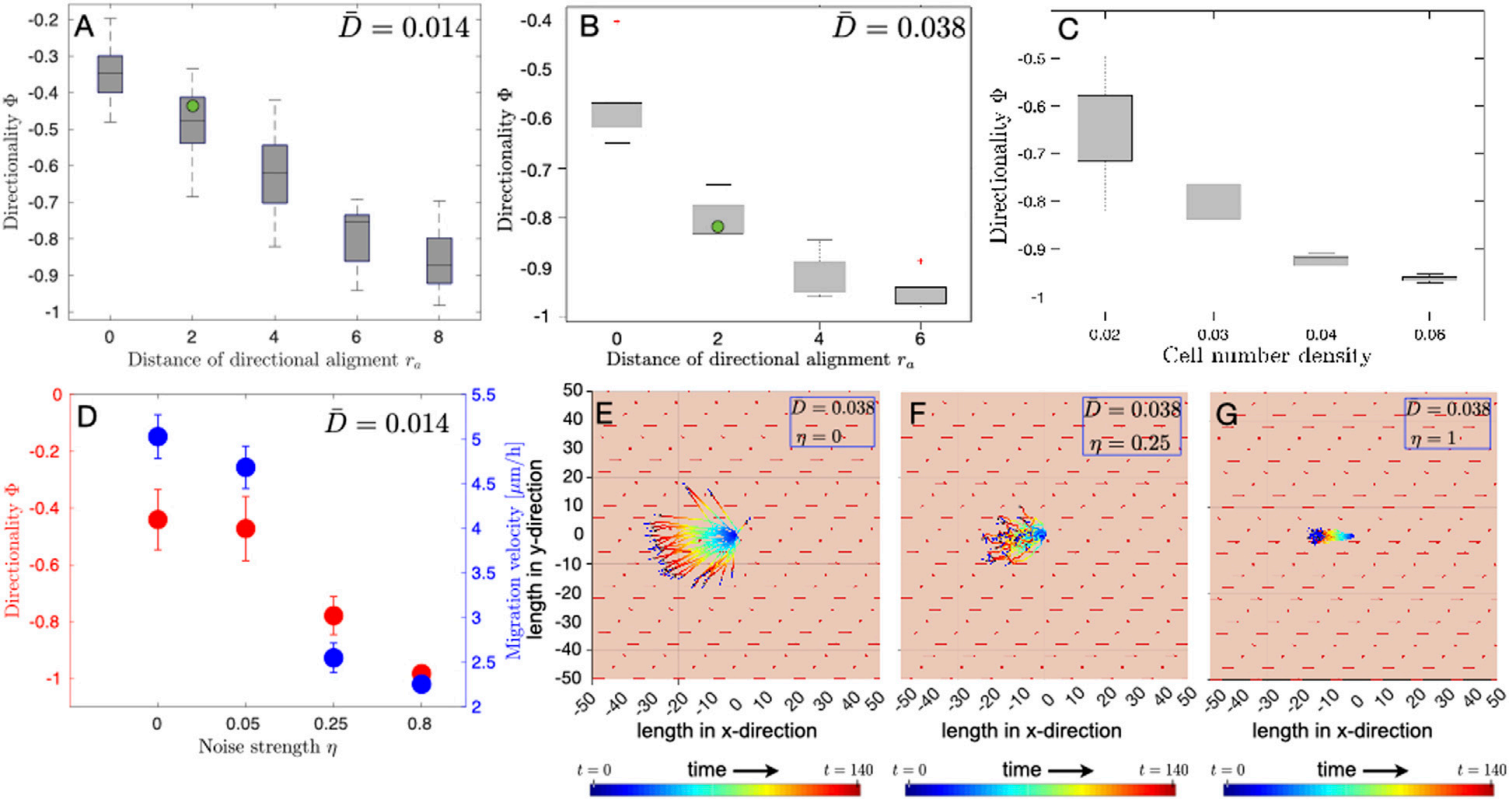
Influence of the radius of alignment, noise strength and cell number density on the directionality of cell migration. A parameter sweep was performed to study the influence of the radius of directional alignment *r*_*a*_ and cell number density on the directionality Φ of electrotactic cell migration of osteoblasts. The green dot in **(A)**, **(B)** is the value of directionality obtained from experiments. Whiskers, in the box and whisker plot **(A–C)**, denote 25–75 percentiles of data distribution. **(A)** Box and whisker plot of medians (horizontal lines) in the case of electrical stimulation of strength $\bar{D}=0.014$ , which corresponds to 160 V/m in experiments. The values of all the other parameters are as mentioned in Table 1. Cells show higher directedness Φ in their migration towards the anode with increasing distance *r*_*a*_ over which directional alignment occurs. Φ = −1 corresponds to fully directed movement towards the anode, which is located at 180° in Figures 1D–I. **(B)** Box and whisker plot of medians (horizontal lines) in the case of electrical stimulation of strength $\bar{D}=0.038$ , which corresponds to 436 V/m in the experiments. The values of all other parameters, except $\bar{D}$ , are as mentioned in Table 1. **(C)** Box and whisker plot of medians (horizontal lines), obtained from simulations, in the case of electrical stimulation of strength $\bar{D}=0.038$ (436 V/m), showing the influence of cell number density on the directionality of cell migration Φ. The values of all other parameters, except $\bar{D}$ , are as mentioned in Table 1. **(D)** Directionality order parameter Φ (red) and migration velocity (blue) vs. noise strength *η*. The values of all other parameters, except *η*, are as mentioned in Table 1. Error bars show the standard deviation in the directionality and the migration velocity for different values of noise strength obtained from simulations. **(E–G)** Final positions and trajectories of individual cells in the case of DC electrical stimulation of strength 0.038 (0.038 corresponds to 436 V/m in experiments). Cell positions are adjusted such that all the trajectories originate from *x* = 0 and *y* = 0 at *t* = 0.

In our model, the collective behavior results from the directional alignment of individual cells with their neighbors. This is controlled by the model parameter *r*_*a*_, the distance over which the cell aligns its direction of migration with its neighbors, and *η*, which is the strength of the fluctuation in the direction of the migration of an individual cell. In the simulation results discussed in the preceding section, Figure 3, we set *r*_*a*_ = 2*R*, which implies the direction alignment of velocity occurs only when cells touch each other. We wondered how the distance over which a cell can align with its neighbors and the noise in the directional sensing influences the collective behavior of osteoblast cell migration. Therefore, we performed a parameter sweep study of the model parameter *r*_*a*_, which determines the distance over which a cell aligns its direction of motility with its neighbours, with fixed electrical stimulation of strengths 0.014 and 0.038, which correspond to 160 V/m and 436 V/m in experiments, respectively. Our results show that even in the case of weak electrical stimulation $\left(\bar{D}=0.014\right)$ , which corresponds to 160 V/m in the experiments, with increasing *r*_*a*_ the cells move in a more directed manner towards the anode, i.e., Φ approaches the value of − 1, as shown in (Figure 4A). This trend, in the dependence of Φ on *r*_*a*_, is also observed when the electric field strength was varied, from $\bar{D}=0.014$ (160 V/m) to $\bar{D}=0.038$ (436 V/m), keeping all the other model parameters same in the two cases (Figure 4B). We also found that by only changing the electric field strength $\bar{D}$ , the model best fits the directionality obtained from experiments for *r*_*a*_ = 2 (Figures 4A,B, where the directionality value obtained from experiments is shown as a green dot). The values of all the other parameters except *r*_*a*_ and $\bar{D}$ are as mentioned in Table 1. This parameter sweep-based analysis, in which only the electric field strength is varied and all the other parameters of the model are kept the same in the two cases, suggests that the applied electric field has the strongest influence on the directionality of osteoblast cell migration, and that the cell-alignment interaction in individual osteoblast cell migration is mainly due to direct contact between the cells. The influence of radius of alignment on the directionality of migration further raised our interest in exploring its possible duality with the cell number density and its influence on the directionality of osteoblast cell migration. The frequency of cell-cell interactions, or contacts, is expected to be higher with increasing cell number density. Using the model, and by performing a parameter sweep analysis, we studied the influence of cell number density on the directionality of cell migration. We considered four different cell densities of 0.02, 0.03, 0.04 and 0.06, each of which correspond to a total number of 25, 35, 45 and 65 cells, respectively. Our results show that the alignment of osteoblast cell migration with the external electric field, and its directedness towards anode increases with cell number density (Figure 4C). For the same values of all the model parameters and an electric field strength $\bar{D}=0.038$ (which corresponds to 436 V/m), our simulation results revealed that the directionality Φ approaches a value of − 1 in the case of cell density of 0.06 (Figure 4C).

#### 3.2.7 Influence of noise on cell migration

Osteoblast cells do not migrate along a straight trajectory over long time intervals. Using our model, we studied the influence of fluctuations in the direction of migration on both the directionality and the effective velocity of electrotactic migration of osteoblasts. Cell movement showed higher directedness with increasing noise strength *η* (Figure 4D). Although cells move in a more directed manner towards the anode, the effective cell migration velocity, on the contrary, decreases with increasing noise strength (Figures 4D–F). The plot of cell trajectories shows that although cells move in an increasingly directed manner towards the anode, they, traverse shorter distances as angular fluctuation increases. These results suggest that the parameters, i.e., *r*_*a*_ and *η*, can significantly alter the dynamics of cell migration and give rise to collective behavior in the electrotactic motion of osteoblast cells.

## 4 Discussion

The migration of osteoblasts, which plays a key role in bone regeneration, can be modulated by external electrical stimulation (Ferrier et al., 1986). This offers an attractive approach toward building electrically active implants for effective tissue regeneration (Brighton et al., 1985; Kaivosoja et al., 2015; Hiemer et al., 2016). In the present paper, we presented a computational model to study (i) the migratory behavior of osteoblasts and, (ii) the consequences of the application of external electrical fields on their migration. The model was used to study the collective behavior of many cells in *in vitro* experiments where primary human osteoblasts placed in an electrotaxis chamber were stimulated by a DC electric field. For this purpose, we re-analyzed the galvanotactic migration of human osteoblasts exposed to DC-electric field stimulation at different field strengths from a previous study published in (Rohde et al., 2019), now using single-cell rather than clustered data. As observed in our previous paper (Rohde et al., 2019), we confirmed that field exposition leads to migratory directionality towards the anode, and elucidate that the migratory speed distribution ranges from 2 to 18 *μ*m/h, with significantly higher speeds of migration than unstimulated cells at DC-field strengths of 300 and 436 V/m. At this point, it remains elusive why there is a significant impact on migration speed at field strengths of 300 and 436 V/m, and not at other stimulation strengths, beyond the general observation that non-linearities do often emerge in biological systems. This point certainly merits further molecular exploration. Using this single-cell analysis approach, beyond our initial findings in the cited paper using pooled data, i.e., stimulated vs. unstimulated only, we show quantitatively that the directionality of cell migration is thus actually influenced by the field strength, with random migration without stimulation, ∼ 65% anodal migration at low (160 V/m) and exclusively anodal migration at highest field strength (436 V/m). Our detailed cell-by-cell analysis also shows that, although the directionality of cell migration clearly correlates with the strength of the applied electric field, there is only a weak correlation between migratory speed and electric field strength, a correlation which could not be seen in the pooled analysis of our previous paper.

To explain these experimental observations, we modeled each cell as an active agent whose movement is influenced by its interactions with other cells, external electric field, and stochastic switching in the direction of migration. The model considers the force experienced by the cell due to the applied DC electric field. We also considered two types of intercellular interactions: in addition to the nearest-neighbor interaction that ensures finite-volume exclusion by penalizing cell overlaps, cells also interact with other cells *via* a velocity alignment mechanism. Although specific molecular mechanisms underlying these interactions remain unclear, two important questions can be addressed by the current simulation study: (i) Does directionality also depend on interaction among neighbouring migrating cells, and if so, how large is this interaction radius, (ii) Do directionality and migration speed depend on the accuracy of the putative cellular field sensing mechanism, i.e., in which way does a noise factor influence migration directionality and migration speed?

Our results show that the motility behavior of cells is influenced by the distance over which the cell aligns with its neighbors, stochastic switching in the direction of migration, and the strength of the applied electric field. The simulations in the present paper closely match the experimentally observed weak correlation between migration speed and the applied electric field and are more realistic than previously published ones (Vanegas-Acosta et al., 2012), which predicted speed ranges from 1.8 to 4.0 *μ*m/s, i.e., nearly tenfold the maximum observed by us. As discussed previously, migration at such high speeds probably finds its limitations in adhesive forces acting on the cells on the one hand and rate-limiting factors such as actin conformational change being limited by temperature and Ca^2+^ dynamics (Sich et al., 2010; Jacobs et al., 2011). We performed a quantitative comparison of the directionality order parameter obtained from simulations with experiments as shown in Figure 3G, where the directionality Φ obtained from experiments practically overlaps with the values obtained from simulations. As the simulation results show, varying *r*_*a*_ from 0 (i.e., the case with no inter-cellular interactions) to 8 (i.e. the case with inter-cellular interactions between two cells extending to distances of four cell diameters), the directionality obtained from our experiments best matches the simulation results in which *r*_*a*_ has the value equal to 2. These results suggest that the interactions between cells only in direct contact likely lead to parallel anodal movement. The mechanism of this interaction could be speculated to rely on e.g., osteoblast binding *via* cadherin, an interaction known to be important for morphogenesis of osteoblasts and subsequent modulation of actin function (Stains and Civitelli, 2005; Stains et al., 2019). Long-distance effects, mediated by e.g. molecules secreted from the cells, tension changes within the collagen coating or distortion of the electric field by the neighboring cell are, in turn, unlikely to be important for osteoblasts. Using our model we also performed a quantitative parameter sweep study to explore the influence of cell number density on the migration directionality parameter Φ. The computational results show that higher cell number density gives rise to higher overall directedness in the cell migration. At higher cell number density one would expect higher number of cell-cell interactions leading to directional alignment of migration, as observed in our simulations.

Our results show that stochastic orientational switching can significantly alter cellular electrotactic motility behavior. In this case, a perfectly directed motion towards the anode is achieved for very high fluctuation strengths, which appears to be counter-intuitive since one would expect that the accuracy of directional movement aligned with the electric field decreases for higher angular fluctuations. Varying *η* in our simulations from 0 to 0.8, the directionality of ∼ −0.45 in our experiments is in line only with a very low degree of noise (around 0.05, which corresponds to fluctuations of ∼10 ° in the direction of cell migration), but not commensurate with values of > 0.25. The experimental migration speed found to be in the range of 2–12 *μ*m/h would also cover the simulated value of ∼ 4.75 *μ*m/h at *η* = 0.05. It is, however, conceivable that other cell types do show more influence of noise (arguably reflecting e.g., less mechanical interactions with the substrate, varying cell shape influences, or different field sensing or signaling mechanisms). What remains to be explained is the seemingly paradoxical result that higher fluctuation levels should lead to higher accuracy in directionality. Our hypothesis would be that higher fluctuation raises the probability of cell-to-cell interactions, which will lead to common field alignment. If this hypothesis holds, such movement will lead to cell alignment of migration direction with the field with higher accuracy but lower speed due to frequent corrective movements. Although experiments are needed to validate this hypothesis, it is interesting to note that at the highest stimulation strength of 436 V/m, those cells which are best aligned to the field and directed towards the anode do not belong to the fastest subset of cells (which are, indeed, 10°–30° off the “ideal” orientation; see Figure 1F.

## 5 Conclusion

The computational model presented here provides a framework for studying *in vitro* DC electrotactic migration of osteoblast cells in two dimensions and elucidating the rules and the role of individual cell interactions with other cells and with their physical environment. This model is also relevant to study the influence of additional factors on cell migration, such as the cell density and other modes of electrical stimulation, e.g. alternating current stimulation. The model we present here allows for easy integration of additional details, as more data becomes available. Our approach could serve as a tool to not only test existing hypotheses of electrotactic cell migration but also predict migratory behaviour under perturbation conditions, and thus bridge the gap between single cell and collective response in a more effective manner.

## Data Availability

The original contributions presented in the study are included in the article/Supplementary Material; further inquiries can be directed to the corresponding authors.
