# Supplementary material for: Cell-cell interactions and fluctuations in the direction of motility promote directed migration of osteoblasts in direct current electrotaxis
Source: Front Bioeng Biotechnol. 2022 Oct 6;10:995326. doi: 10.3389/fbioe.2022.995326 (PMC9582662; doi:10.3389/fbioe.2022.995326)
Supplement: Supplementary file 1 [file DataSheet1.PDF]

# Supplementary Material

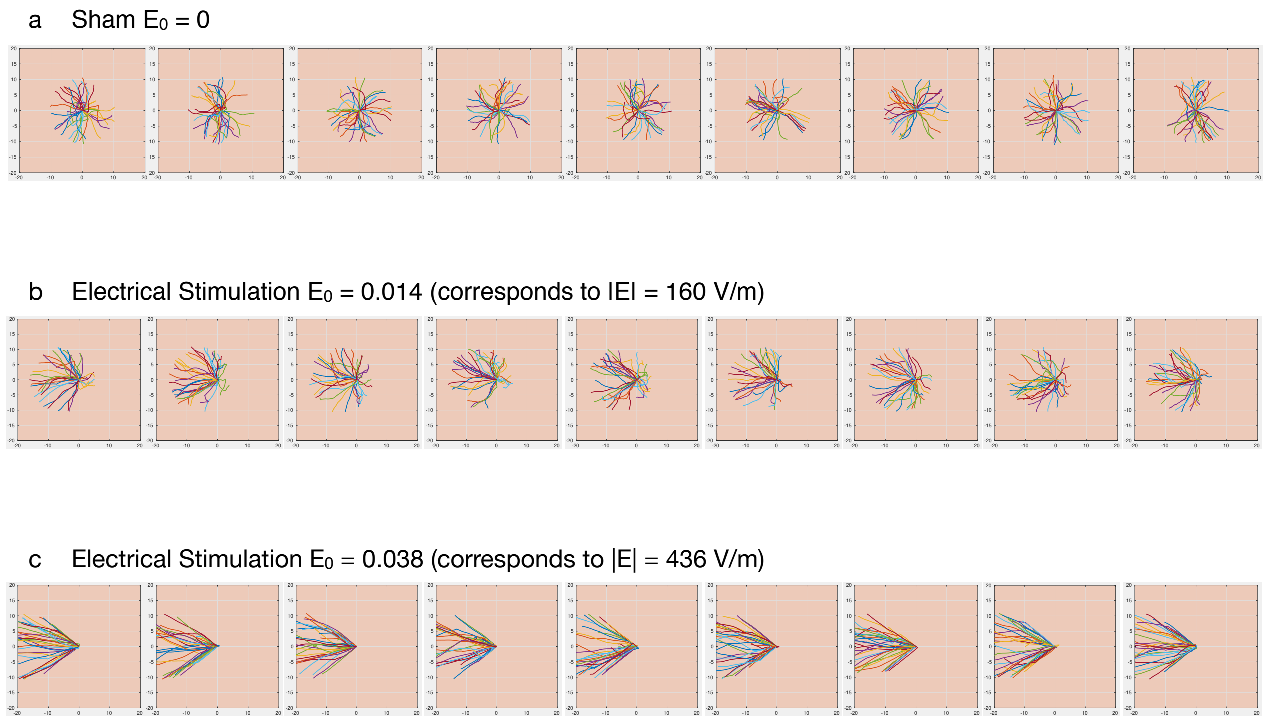


**Figure S1. Individual cell trajectories for multiple simulation runs.** Results of ten separate simulation runs for the case: (A) no electrical stimulation, which corresponds to experimental sham, (B) stimulation with electrical field strength of 0.014, which corresponds to the experimental field stimulation strength of 160 V/m, and, (C) stimulation with electrical field strength of 0.038, which corresponds to the experimental field stimulation strength of 436 V/m.
